# Effect of a Visual Dual-Task on Single-Leg Countermovement-Jump in Male Professional Soccer Players with Lower-Limb Injuries: A Cross-Sectional Observational Study

**DOI:** 10.3390/sports13120419

**Published:** 2025-12-01

**Authors:** Geoffrey Memain, Christopher Carling, Jean Bouvet, Paul Fourcade, Pascal Maille, Eric Yiou

**Affiliations:** 1 FIFA Clairefontaine Medical Center and Research Centre, French Football Federation, 78120 Clairefontaine-en-Yvelines, France; ccarling@fff.fr (C.C.); jbouvet@fff.fr (J.B.); pmaille@fff.fr (P.M.); 2 CIAMS Laboratory, Université Paris-Saclay, 91400 Orsay, France; paul.fourcade@universite-paris-saclay.fr (P.F.); eric.yiou@universite-paris-saclay.fr (E.Y.); 3 CIAMS Laboratory, University of Orléans, 45100 Orléans, France; 4 Laboratory Sport, Expertise and Performance (EA 7370), French Institute of Sport (INSEP), 75012 Paris, France

**Keywords:** countermovement jump, sport rehabilitation, dual task, high-level soccer, lower-limb injuries

## Abstract

**Background:** Participation in soccer imposes high physical, mechanical, and cognitive demands. Recent evidence suggests that cognitive load, often overlooked in injury prevention, interacts with biomechanical factors and injury risk, resembling a dual-task paradigm where players must adapt motor responses while processing unpredictable game situations. This cross-sectional observational study examined how adding a dual-task during single-leg countermovement-jumps (SLCMJ) affects neuromotor control and performance in elite soccer players. **Methods:** Players performed SLCMJ on the injured leg while muscle activation, kinematics, and kinetics were measured, with and without a dual-task requiring color identification, via repeated-measures ANOVA; three injured groups (Chondropathy, n = 10, ACL, n = 15, Muscle Injury, n = 15) and a healthy control group (n = 22, followed the same protocol during final-rehabilitation stage. **Results:** Specific main outcomes were kinetics, kinematics, and EMG variables. Kinetic performances were significantly higher (*p* < 0.001, d > 0.6) with dual-task: eccentric rate-of-force-development, jump-height, reactive-strength-index-modified, and shorter for time-to-peak of ground-reaction-force (*p* < 0.05, d > 0.6). Muscle activation increased with dual-task in rectus femoris and biceps femoris during pushing (eccentric and concentric phases) (*p* < 0.01, d = 0.7) and for medial gastrocnemius during landing (*p* < 0.05, d = 0.7). Kinematic analyses showed greater pushing knee flexion, while pushing and landing trunk flexion was lower (*p* < 0.01, d > 0.8). Kinetic values in the three injured groups were lower than those of controls (*p* < 0.01, d > 0.8). **Conclusions:** Injured elite soccer players appeared disinhibited in dual-task conditions that improved SLCMJ performance but altered neuromotor control, underscoring the importance of a neurocognitive approach in return-to-play assessments to evaluate reinjury risk.

## 1. Introduction

Participation in soccer places substantial physiological, mechanical, and cognitive demands on players [1]. Arguably, the cognitive dimension of the game has been poorly taken into consideration in current injury prevention and reconditioning programs. Soccer players are frequently confronted with unpredictable [1] game situations requiring rapid visual analysis of the surrounding environment. The most studied factor in the field of propulsion, landing, and injury risk is fatigue [2,3]. However, a recent systematic review highlights the association between cognitive demands, biomechanical indicators, and injury risk [4]. These situations necessitate adaptation of players’ motor responses to the ongoing play while simultaneously moving at high velocities, and can be assimilated to a “dual-task” paradigm [5], where participants perform simultaneously a primary and a secondary task. The first is a motor task (such as running, dribbling, kicking, etc.) while the second can be visual in nature (such as gazing at other players’ positions on the field). Dual-task paradigms are classically used in the literature to investigate the attentional cost devoted to the primary task, with the hypotheses that participants divide their attentional resources between the primary and the secondary task at the same time, and that the total amount of attentional resource capacity of humans is limited [6]. As such, if the attentional demands of the primary task are high, the superimposition of the secondary task might lead to an alteration in the performance of one or both tasks.

In the dual task paradigm, the secondary task is assimilated to a “probe” directed to reveal the attentional cost of the primary task [5]. It has been suggested that a negative effect of the probe is its propensity to increase the risk of injury when a locomotor task is incorporated in the dual task [7]. For example, alterations in kinematic patterns during a jump landing task (primary task) are characterized by lower knee flexion and higher knee abduction [8,9,10] in addition to modified kinetic patterns during a countermovement jump, characterized by a higher peak of vertical ground reaction force during landing [8], have been reported when visual information uptake (the secondary task) is added to the primary task. Furthermore, alterations of variables such as GRFz and trunk flexion, trunk lateral flexion, and knee flexion during the landing phase of a jump appear to be predictive of anterior cruciate ligament (ACL) injury, with a specificity of 73% and a sensitivity of 78% [11]. Moreover, ankle mobility and foot stability have a key role in these jump receptions [12]. Modification of the electrical activity of the lower limb muscles, characterized by increased biceps femoris and medial gastrocnemius muscle activities, notably, along with decreased activity in the rectus femoris and vastus lateralis, has also been reported during jump landing or cutting maneuvers performed under similar dual task conditions [1]. These changes in locomotor patterns have been described as potential sources for knee injury [11] and typically chondropathy and ACL ruptures [10], two injuries commonly incurring in soccer.

However, a recent meta-analysis by Salihu et al. [13] demonstrated that motor alterations under dual-task situations were not systematic in motor tasks where there is a postural component (which is the case for virtually all sport-related tasks). The authors stressed that only 50% of the studies they analyzed reported an alteration in postural performance under a dual-task situation, while 20% did not report any significant effect, and 30% even reported an improvement. According to the authors, these inconsistent results might be related to the difficulty of the postural task challenge. In general, the higher the postural task challenge, the higher is the attentional demand and the subsequent alteration of the performance [9].

Through intensive sport practice, it is recognized that specific motor routines become automatized and less challenging [10], i.e., they can be performed with less attentional cost (at times, if any), which allows more room for the development of game strategies. In comparison to beginner and athletes competing at lower levels, it is logical that those performing in high-level sport are expected to be less sensitive to the negative effects of dual-tasking, at least when performing routine sport movements [14]. Additionally, the neurocognitive approach is arguably still one of the major missing components in prevention programs [15]. However, to our knowledge, no information exists on whether the level of automation and related attentional cost required for these motor routines are modified in high-level athletes when they are injured. As such, it is important that medical and reconditioning staff also evaluate the cognitive dimension of movement to help ensure safe return-to-play. Indeed, injured athletes might employ a strategy where they increase their level of attention during movement execution to attain a similar level of motor performance as healthy non-injured peers [14]. Such protective cognitive strategies might be masked when the motor routines included in the reconditioning program are evaluated in a single-task paradigm. Consequently, the risk of re-injury during the return-to-play phase could then be underestimated by medical staff.

The aim of this study was to evaluate the level of motor automation (the ability to produce a movement with reduced requirement for conscious attention, lowered cognitive load, and increased efficiency and consistency of movement control [16]) and neuromotor alteration of a typical sport routine movement, in dual-task, in injured professional soccer players compared with healthy peers. For this purpose, the effect of a visual information scanning task (secondary task) on motor patterns during a single-leg countermovement-jump (SLCMJ, primary task) was examined in healthy male professional soccer players and in those who had sustained a lower-limb injury. Players suffering from the three most frequent injuries encountered in soccer participated: those with an ACL rupture, a chondropathy, and a muscular lesion in the hamstrings or quadriceps. It was hypothesized that the level of attention devoted to this task would be higher in injured athletes compared to controls, as revealed by a greater negative effect of the dual-tasking on the motor patterns of the primary task (kinematics, kinetics, and muscle activity). Second, the magnitude of this effect might be dependent on the type of injury. The primary outcome measure is mainly the evolution of kinetic performance data, but also the behavior of kinematic data and muscle activation levels.

## 2. Materials and Methods

### 2.1. Study Design

**Figure 1:**
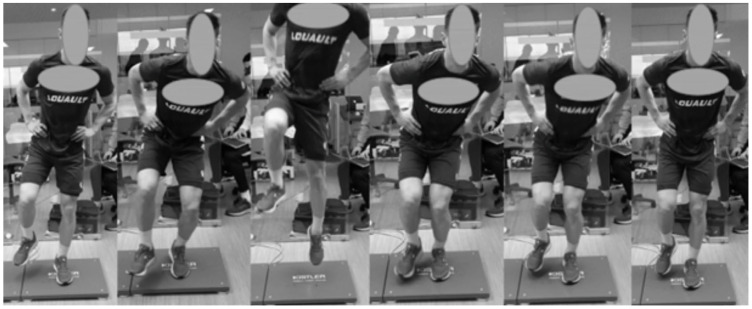
Single-leg countermovement-jump of an injured player during single and dual-task conditions.

This study is an observational, cross-sectional laboratory study (See Supplementary Materials). The protocol was conducted between 2:00 and 3:30 PM in the biomechanics laboratory of the Clairefontaine National Football Center (Yvelines, France). The temperature of the room was set at 20 °C. The protocol systematically commenced with a warm-up of 10 min on a cycle ergometer, with the power gradually increasing from 100 W to 200 W. For each leg muscle, two successive evaluations of the maximal isometric voluntary contraction (MIVC) [17] were performed using the appropriate materials and postures (see paragraph “EMG normalization”). A series of SLCMJ was then performed under a single or a dual-task condition (see paragraph “Study design” for description). In the initial posture, participants stood upright on a force-plate with their injured leg (for the injured groups) or their dominant leg (for the control participants), and with their hands on their hips (Figure 1). The contralateral leg was slightly flexed, with the foot placed a few centimeters above the force plate. In both the single and dual-task conditions, participants were instructed to jump as fast and as high as possible with self-paced, and to land on the same leg on the force-plate. They had to maintain their final posture (the same as the initial posture) for a few seconds before they could rest. The SLCMJ is a task that many soccer players perform every day during their routine warm-up.

In each condition, five successive SLCMJ were performed. The first two trials were blank (results not recorded). These were performed to ensure the participants’ familiarity with the experimental apparatus and instructions. Single and dual-task conditions were randomized across participants to avoid rank effects and separated by a 1 min rest period to avoid any potential effect of fatigue. A pilot study conducted by the present authors in healthy athletes (n = 22) showed that there was no statistical effect for leg dominance on the experimental variables of the present study. Any between-group differences observed for these variables could therefore not be ascribed to an effect of leg dominance.

In the single-task condition, SLCMJ were performed in isolation under the conditions described above. In the dual-task condition, SLCMJ (primary task) was performed in combination with a simultaneous uptake of visual information (secondary task). In this secondary task, participants had to declare aloud and as quickly as possible the color (among four choices: white, red, blue, yellow) displayed on an electronic pod placed 3 m away in front of them and at eye level. The display time of the pod was randomized during the jump. The experimentation was performed in a block paradigm.

### 2.2. Setting

All participants played in one of the professional soccer leagues in Europe. They were recruited by the doctor, co-author of the study, between 2 June 2020 and 2 December 2022 during their rehabilitation stay and during pre-season preventive testing at the FIFA FFF Clairefontaine Medical Center.

### 2.3. Participants

A total of sixty-two participants were enrolled in the present study. All were male professional soccer players who were either healthy control group (CT, n = 22) or had sustained a lower-limb injury (n = 40). The justification for the small sample size primarily relates to resource limitations [18], including both time constraints and the rarity of the studied population—professional football players, the specific type of pathology, and the timing of assessment during sports rehabilitation—which together represent significant challenges to recruiting a large sample. Twenty-two players were injured on their dominant leg versus 18 injured on their non-dominant leg. Their positions on the pitch are varied, but none of them are goalkeepers. All participants played in one of the professional soccer leagues in Europe. The Injured participants were included in one of the three following groups: chondropathy (CH group, n = 10), quadriceps and hamstring injuries (MI group, n = 15), and Anterior Cruciate Ligament rupture (ACL group, n = 15). Participants were in the “on-field rehabilitation” final phase [19,20,21]. Inclusion criteria in the injured participants’ cohort included being able to run for 30 min at a moderate pace and hop on one leg, all without pain. Participants in the healthy control group were included if they were adult male professional soccer players without a significant injury (light severity < 8 days of absence from training [22]) of the lower-limb during the 6-month period preceding the protocol. The time elapsed since the injury and the type of graft used for the ACL are not factors that we consider as major, as what is more important is the phase of treatment and the functional validation of the athlete’s abilities [23,24]. Furthermore, the type of graft used for ACL reconstruction has little influence on rehabilitation and RTP [25]. The anthropometric features of the participants are reported in Table 1. This study complied with the Declaration of Helsinki (1964), and permission was obtained from the French national ethics committee for sports science research (CERSTAPS n°IRB00012476-2020-24-03-48).

### 2.4. Variables

**Figure 2:**
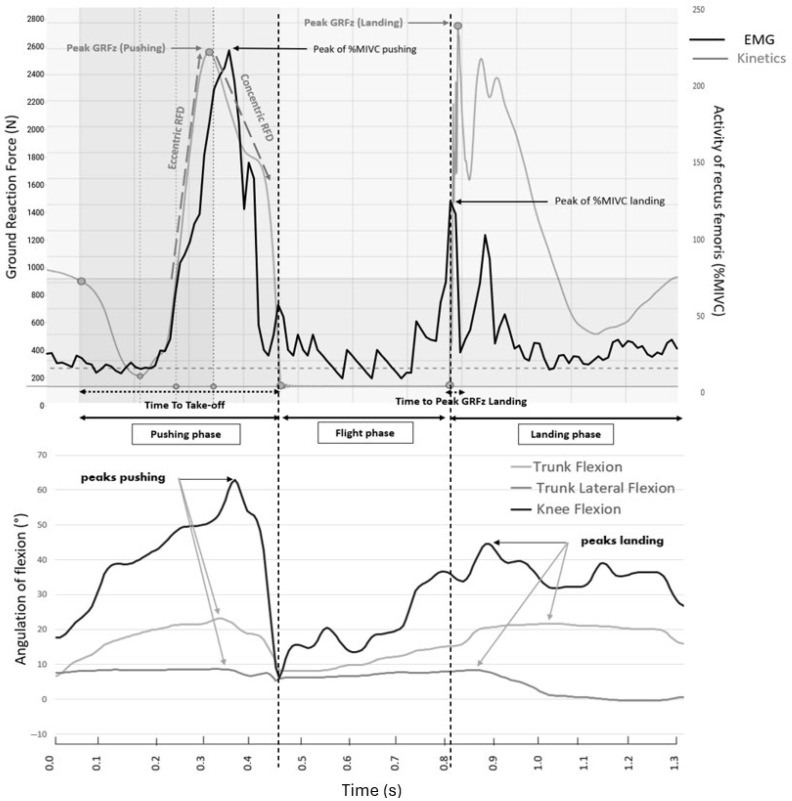
Typical biomechanical traces of a single-leg countermovement-jump showing selected experimental variables (one representative participant of the healthy control group). Variables: RFD: Rate-of-force development, GRFz: ground reaction force in *z*-axis, %MIVC: percentage maximal isometric voluntary contraction.

The following spatio-temporal and kinetic variables were obtained from the force-plate analysis (Figure 2):

− Duration of the pushing phase or time to take-off (in ms), which corresponds to the delay between the onset variation in the vertical ground reaction force (GRFz) and the stance foot take-off.
− Jump height (in cm), which corresponds to the maximal height reached by the athlete during the SLCMJ. This variable is estimated by the force-plate software (Mars 3.0, Kistler, Winterthur, Switzerland) from the flight time and reflects the athlete’s neuromotor functional performance [17].
− Reactive Strength Index Modified (RSI-mod, in m/s), which corresponds to the ratio between jump height and time to take-off. This variable reflects the lower-limb explosiveness [17].
− Rate-of-force development [17] (RFD, in N/s) during the eccentric (RFD_eccentric_) and the concentric (RFD_concentric_) period of the pushing phase (Figure 2). RFD_eccentric_ corresponded to the line of slope passing through to the athletes body weight return (same level as before the beginning of the CMJ, the first point of the kinetic curve on Figure 2) when climbing the GRF and the first upward peak of the vertical ground reaction force trace; RFD_concentric_ corresponded to the slope of the line passing through this first upward peak and the time of take-off [26]. These two RFD values further reflected lower-limb explosiveness. RFD values are strongly related to vertical jump performance [17].
− Ground reaction force in *z*-axis (GRFz) value at t = 50 ms after the foot landing (in Newton). The risk of knee injury is known to be maximal at this instant [27].
− Time to peak GRFz during the landing phase (in ms), which corresponds to the delay between the foot landing and the peak of GRFz.

EMG variables included the peak and mean values of the leg muscle electrical activity (expressed in percentage of the activity obtained during the maximal isometric voluntary contraction) during both phases of the SLCMJ (Figure 2) [28].

The kinematic variables included the peak of knee flexion, the peak of trunk flexion, and the peak of trunk lateroflexion (in degrees) obtained during both phases of the SLCMJ [29] (Figure 2).

### 2.5. Data Recordings

Movement analyses included electromyographical (EMG), kinematic, and kinetic data collection. Kinetic data were recorded with a force plate (9260AA6, 600 × 500 mm, Kistler Instruments, Hampshire, UK), which displayed the ground reaction forces and the moments acting at its surface. Only the vertical component of the ground reaction force vector was analyzed. Kinematic data for the stance knee (the leg that propels the body upward) and the trunk were recorded using the Humantrak system (Vald Performance, Brisbane, Australia) equipped with one Kinect v2 camera (Microsoft Corp., Redmond, WA, USA). Residual noise was eliminated by a dual Butterworth filter. The electrical activity of the following stance leg muscles was recorded with a 12-channel Delsys Trigno equipped with wireless surface Ag/AgCl sensors (27 mm × 35 mm, Trinoma, Lyon, France): biceps femoris, semitendinosus, vastus medialis, rectus femoris, gluteus medius, and medial gastrocnemius. SENIAM recommendations were followed to determine the location of the electrodes on the muscles [30]. The Butterworth filter used was a bandpass filter that suppresses out-of-range noise (10–450 Hz) with effective attenuation (40/80 dB per decade). A 10–450 Hz bandpass filter was used to filter the EMG signals in EMGworks 4.4 software (Delsys, Inc., Manchester, UK) by Root Mean Square [31]. The sample frequency was set at 1000 Hz for the EMG and kinetic recordings, and at 30 Hz for the kinematic recordings.

### 2.6. EMG Normalization

**Figure 3:**
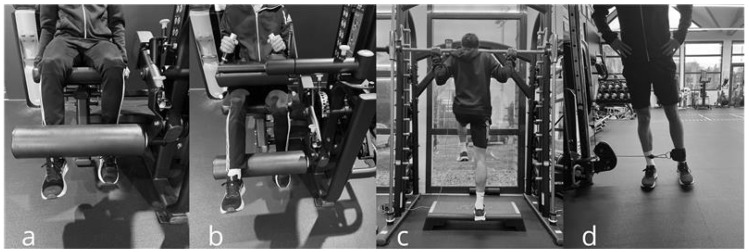
Standardized conditions to evaluate the maximal isometric voluntary contraction of leg muscles with 2 repetitions of 5″: (**a**): the vastus medialis and rectus femoris on leg extension; (**b**): semitendinosus and the biceps femoris on leg curl; (**c**): medial gastrocnemius on calf press; (**d**): gluteus medius on abduction at fixed pulley.

The activation level of the leg muscles during the SLCMJ was normalized with respect to their activation level recorded during maximal isometric voluntary contractions. To determine this maximal activation, two successive 5 s repetitions of maximal contractions were performed with a 30 s recovery interval [30]. The highest 0.5 s sliding average was taken as the maximal isometric voluntary contraction [30]. This reference value was measured on a guided machine during specific analytical exercises against over-maximal resistance: leg extension at 45° knee extension for the rectus femoris and vastus medialis, 45° flexion on leg curl in support of biceps femoris and semitendinosus, 25° hip abduction standing leg on fixed pulley in support of gluteus medius, and knee extension on calf press with the foot placed in neutral ankle flexion for the medial gastrocnemius [31] (Figure 3).

### 2.7. Description of the Biomechanical Traces (Figure 2)

In the kinetic trace, a decline is observed matching the countermovement phase, marking the release of the athlete’s weight. Subsequently, the trace ascends during the reloading phase as the eccentric movement concludes, reaching its peak. Following this, another decline occurs during the concentric thrust phase, succeeded by a brief plateau, denoted by zero values in the kinetic trace, representing the time of flight. Finally, the trace ascends once more during the landing phase of the jump before reaching another plateau.

### 2.8. Bias

Sampling during recruitment is not random, given the specific characteristics of the population studied. It is difficult to generalize the results given the specific audience studied. The kinematic assessment tool is limited to 30 Hz.

### 2.9. Study Size

A sample size estimate was not performed prior to recruitment due to practical constraints related to the exploratory nature of the study and the rarity of the target population (professional footballers in the final stages of rehabilitation). For information purposes, we performed post hoc power calculations to illustrate the sensitivity of our work. Assuming a one-factor ANOVA test (α = 0.05, desired power 80%) and four groups (CH, MI, ACL, CT), the required sample sizes would be: approximately n ≈ 274 per group for a small effect, n ≈ 45 per group for a medium effect, and n ≈ 23 per group for an effect size close to that observed in our analyses. Our study is therefore informative for detecting moderate to large effects but underpowered for detecting small to moderate effects. This limitation is discussed in the Limitations section, and we recommend that future studies aim for more than 25 participants per group (a total ≈ of 100) to achieve greater power.

### 2.10. Statistics Methods

The data follow a normal distribution according to a Shapiro–Wilk test (*p* > 0.05), and the homogeneity of variances was confirmed by a Levene test (*p* > 0.05). Mean values and standard deviations were calculated for each experimental variable. A repeated-measures ANOVA model with the injury condition (2 levels: single task vs. dual-task) as a within-factor, and the group (4 levels: CH, ACL, MI, and CT) as a between-subject factor was used. To eliminate any effect of player age and expertise on results, a repeated-measures ANOVA was also used to test anthropometric data and years spent in the professional league with the group as a between-subject factor. The Tukey post hoc test was used when a significant main effect was observed. The significance threshold was set at *p* < 0.05. Cohen’s d represented effect sizes which were classed as trivial (<0.2), small (0.2–0.49), medium (0.5–0.79), and large (≥0.8).

No specific sensitivity analysis or adjustment for covariates was performed. This decision was based on several methodological considerations: (1) the study population was broadly homogeneous, consisting exclusively of professional footballers training in the same environment, which limited the main potential sources of confusion (age, training load, level of play); (2) the groups had similar characteristics, making it unnecessary to add covariates; (3) the limited sample size did not allow for the inclusion of multiple covariates without risking a decrease in statistical power or model instability.

Furthermore, seven attempts were canceled due to failure to complete the task (free leg touching the ground, stepping off the platform), and the main analyses revealed consistent trends between the different variables measured, demonstrating satisfactory internal robustness of the results.

Given the descriptive and exploratory nature of the study, additional sensitivity analyses were not deemed necessary at this stage.

## 3. Results

### 3.1. Effect of Group on the Anthropometric Features of Players and Years as a Professional Player

The anthropometric features and years spent in a professional league of athletes in each group are reported in Table 1. No statistical group effect was observed for any of the variables. No participants were excluded from the pathological groups, and eight from the control group, due to injuries resulting in more than eight days of absence from training.

### 3.2. Effect of Condition and Group on Biomechanical Kinetics Features of the SLCMJ (Appendix A)

There was no significant condition X group interaction for any of the kinetic variables during the single versus dual-task condition in all groups.

**Figure 4:**
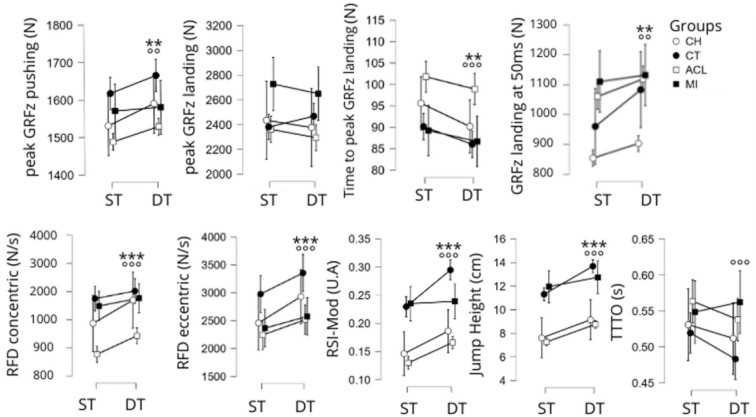
Main effects of the condition and the group on the kinetics variables of the SLCMJ: significant main effect of the condition (single task vs. dual task) with *p* < 0.01 = ** and *p* < 0.001 = ***; significant main effect of the group with *p* < 0.01 = °° and *p* < 0.001 = °°°. ST: Single task; DT: dual task. Groups: CH: chondropathy, CT: control, ACL: anterior cruciate ligament, MI: muscle injury. GRFz: ground reaction force in *z*-axis, RFD: rate-of-force development, RSI: reactive-strength-index, TTTO: time to take-off.

A significant main effect for condition was observed for the following variables: RFD_eccentric_ (F [1,179] = 6.9, *p* < 0.001), RFD_concentric_ (F [1,179] = 6.9, *p* < 0.001), RSI-Mod (F [1,179] = 17.9, *p* < 0.001), jump height (F [1,179] = 20.3, *p* < 0.001), GRFz at t = 50 ms (F [1,179] = 5.8, *p* < 0.05) and time to peak GRFz during the landing phase (F [1,179] = 5.3, *p* < 0.05, Figure 4). With the exception of the last variable and the time to take off (both of which decrease), values for all variables increase from the single-task to the dual-task condition (Figure 4).

There was also a significant main group effect for the following variables: RFD_eccentric_ (F [3,179] = 6.6, *p* < 0.001), RFD_concentric_ (F [3,179] = 7.6, *p* < 0.001), RSI-Mod (F [3,179] = 30.5, *p* < 0.001), jump height (F [3,179] = 27.9, *p* < 0.001), time to take off (F [3,179] = 4.1, *p* < 0.01), peak of GRFz during both the pushing phase (F [3,179] = 4.5, *p* < 0.01) and the landing phase (F [3,179] = 4.6, *p* < 0.01), GRFz at t = 50 ms (F [3,179] = 12.0, *p* < 0.001) and time to peak GRFz during the landing phase (F [3,179] = 7.6, *p* < 0.001). Statistically, RFD_concentric_ was lower in the ACL group than in the healthy control group (*p* < 0.05, d = 0.7) while RSI-Mod was lower in the CH group than in the healthy control group (*p* < 0.001, d = 1.0). Jump height was significantly lower in the MI group than in both the healthy control group (*p* < 0.001, d = 1.1) and the MI group (*p* < 0.01, d = 1.0). Time to peak vertical ground reaction force during the landing was significantly higher in the ACL group than in both the healthy control group (*p* < 0.05, d = 0.6) and the MI group (*p* < 0.05, d = 0.7).

### 3.3. Effect of Condition and Group on Biomechanical Kinematics Features of the SLCMJ

**Figure 5:**
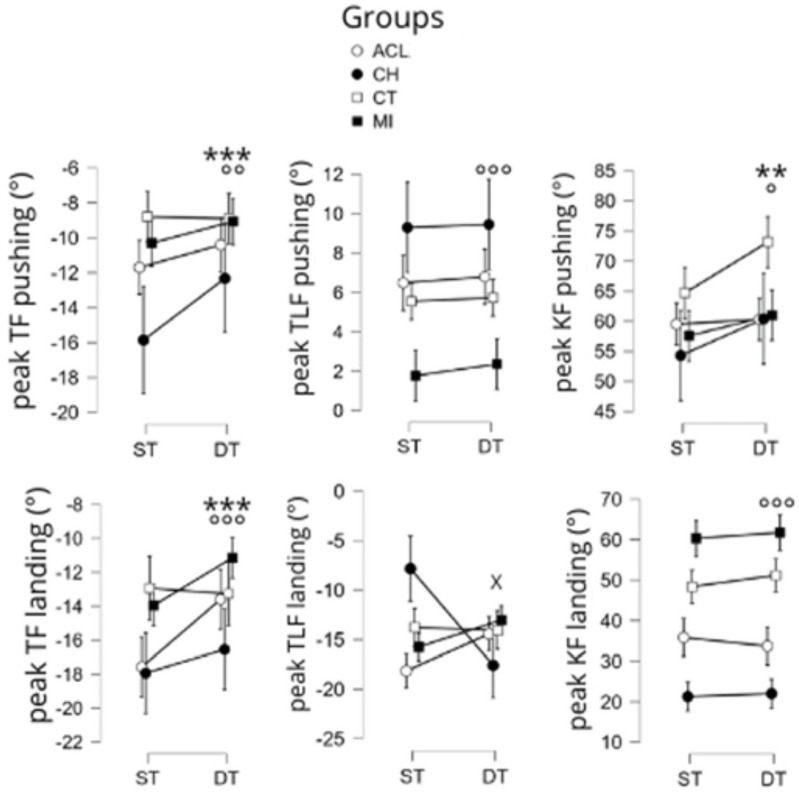
Main effects of the condition, the group, and the group X condition interaction on the kinematic variables of the SLCMJ. Significant main effect of the condition (ST: single task vs. DT: dual task) with *p* < 0.01 = ** and *p* < 0.001 = ***. Significant main effect of the group with *p* < 0.05 = °, *p* < 0.01 = °°, and *p* < 0.001 = °°°. Significant main effect DT X Group with *p* < 0.05 = x. TF: Trunk Flexion; TLF: trunk lateral flexion; KF: knee flexion. Groups: ACL: anterior cruciate ligament, CH: chondropathy, CT: control, MI: muscle injury. The negative values of trunk lateral flexion landing correspond to lateral flexion of the trunk towards the contralateral side (the side opposite to the leg performing the countermovement-jump movement).

There was a significant condition X group interaction on the peak of trunk lateroflexion during the landing phase (F [1,180] = 13.3, *p* < 0.001). Post hoc testing revealed that this interaction was explained by a decrease in values from the single-task to the dual-task condition in the CH group (cf. Figure 5; *p* < 0.001, d = 1.2).

A significant main effect of the experimental condition was observed for the following variables: peak of knee flexion increased during the pushing phase (F [1,179] = 7.5, *p* < 0.01), whereas peak of trunk flexion during both the pushing (F [1,179] = 5.6, *p* < 0.01) and landing phase (F [1,180] = 8.3, *p* < 0.01) decreased.

There was also a significant main group effect on the following variables: peak of knee flexion (F [3,179] = 4.6, *p* < 0.01), peak of trunk lateroflexion (F [3,179] = 7.7, *p* < 0.001), peak of trunk flexion (F [3,179] = 6.1, *p* < 0.001), all during the pushing phase, and peak of trunk flexion during the landing phase (F [3,179] = 5.9, *p* < 0.001). More specifically, this latter variable was statistically lower in the MI group versus in both the ACL group (*p* < 0.05, d = 0.8) and the CH group (*p* < 0.001, d = 1.2), and higher in the CH group versus the healthy group control (*p* < 0.01, d = 0.8). Peak of trunk lateroflexion during pushing was significantly higher in the CH group than in MI peers (*p* < 0.01, d = 1.0).

### 3.4. Effect of Condition and Group on Muscle Activity of the SLCMJ

**Figure 6:**
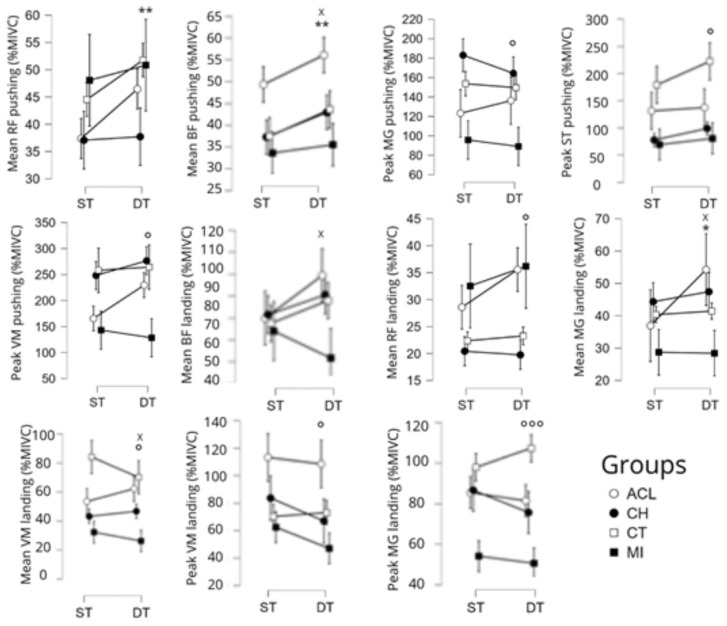
Main effects of the condition, the group, and group X condition interaction on the electromyographical variables of the SLCMJ (expressed in % of maximal voluntary isometric contraction [MIVC]). Significant main effect of the condition (ST: single task vs. DT: dual task) with *p* < 0.05 = * and *p* < 0.01 = **. Significant main effect of the group with *p* < 0.05 = ° and *p* < 0.001 = °°°. Significant main effect DT X group with *p* < 0.05 = x. Groups: ACL: anterior cruciate ligament, CH: chondropathy, CT: control, MI: muscle injury. RF: rectus femoris, BF: biceps femoris, MG: medial gastrocnemius, ST: semitendinosus, VM: vastus medialis.

There was a significant condition X group interaction on the mean activity of the biceps femoris during the pushing phase (F [3,179] = 3.0, *p* < 0.05), on the mean activity of the medial gastrocnemius (F [3,179] = 2.9, *p* < 0.05) and the biceps femoris (F [3,179] = 3.0, *p* < 0.05), and on the peak activity of the vastus medialis (F [3,179] = 3.0, *p* < 0.05) during the landing phase (Figure 6). Post hoc tests showed that the mean activity of the biceps femoris during the pushing phase was statistically higher in the ACL group than in the MI group in the dual-task condition only (*p* < 0.05, d = 0.7), and that, 1) the mean activity of the medial gastrocnemius (*p* < 0.05, d = 0.7), biceps femoris (*p* < 0.05, d = 0.7) and the peak activity of the vastus medialis (*p* < 0.05, d = 0.7) during the landing phase, were all significantly higher in the ACL group than in the MI group in the dual-task only.

There was a significant main effect for the condition on the mean activity of the rectus femoris (F [1,179] = 6.8, *p* < 0.01) and the biceps femoris (F [1,179] = 9.5, *p* < 0.01) during the pushing phase, and on the mean activity of the medial gastrocnemius during the landing phase (F [1,179] = 4.6, *p* < 0.05). These variables increased significantly from the single-task condition to the dual-task condition (Figure 6).

There was also a significant main group effect on the peak activity of the three following muscles during the pushing phase: vastus medialis (F [3,179] = 3.0, *p* < 0.05), medial gastrocnemius (F [3,179] = 3.8, *p* < 0.05), and semitendinosus (F [3,179] = 3.1, *p* < 0.05). During the landing phase, there was also a significant main effect of the group on the mean activity of the rectus femoris (F [3,179] = 3.6, *p* < 0.05) and vastus medialis (F [3,179] = 2.8, *p* < 0.05), and on the peak activity of the medial gastrocnemius (F [3,179] = 6.0, *p* < 0.001) and vastus medialis (F [3,179] = 4.1, *p* < 0.01). Post hoc tests revealed that the MI group had lower peak activity for the semitendinosus than control peers during landing (*p* < 0.01, d = 0.7).

## 4. Discussion

This study investigated the level of motor automation and neuromotor alterations during a typical routine sport movement during a dual-task in professional soccer players with various knee injuries, and compared these with a group of healthy peers. Main findings were that the dual task generated kinematic and muscular activation alterations during a CMJ. However, a positive effect of the dual-task was observed on the kinetic variables, which may be linked to the players’ disinhibition and defocusing from previous fears and discomfort related to their injury.

### 4.1. Dual-Task Impact on Kinetic Variables

Previous research in healthy athletes across a range of sports has generally reported a decrease in performance (jump height, accuracy, reaction time, or speed movement) during different dual-task sport situations [32]. Studies have also reported an increase in peak GRFz and a decrease in stability in dual-task situations during both landing [10] and cutting situations [33]. An increase in peak GRFz is commonly associated with an augmentation of the mechanical stress on the knees, thus increasing the risk of ACL injury [10]. Interestingly, the present results contrast with these previous observations on the impact of a dual-task on pushing and the resulting time to take off (jump height). Indeed, the dual-task seemed to have a “disinhibiting effect” on movement during an SL-CMJ. Values for nearly all variables increased under the DT condition, except peak GRFz and the time to peak GRFz during landing, and the TTTO, which decreased in the dual-task condition. As such, these changes can be considered to have facilitated the SL-CMJ movement. More specifically, results showed that pushing performance was improved in the dual-task condition by increasing values for RFD_concentric_, RFD_eccentric_, RSI-Mod, jump height, and time to peak of GRFz during landing. These results align with previous findings [9], which also demonstrated improvements in performance with the addition of a dual-task, but with alteration of neuromotor control. The positive effect of adding a visual task may be due to the players defocusing their attention from the primary visual task to the secondary task, in addition to the apprehension of old pain. Furthermore, the research findings of Ricupito et al. [34] on the unweighted phase of the CMJ support the benefits of adding dual tasks to sports rehabilitation. These authors observed impaired neuromuscular control and compensatory strategies via lower negative velocity and center of mass displacement, as well as higher minimal GRFz in subjects who had undergone ACL reconstruction [34]. The use of dual tasks in rehabilitation training could help develop the neuromuscular qualities needed to regulate these impairments.

### 4.2. Dual-Task Impact on Kinematic Variables

Previous work [10] suggests that performing a jump landing in a dual-task condition generates a high cognitive load and modifies the biomechanical aspect of the movement, increasing the mechanical load on the ACL [10]. It has also been commonly reported that the addition of a dual-task in healthy recreational athletes is associated with an increase in dynamic knee valgus [8] particularly in the first 50 ms following ground contact. During this dual-task situation, lower knee and hip flexion during both the pushing and the landing phase have been observed [8]. The present results partly contradict these previous findings, as knee flexion during the pushing phase was higher in the dual-task in both the injured and healthy control groups. An increase in knee flexion enables greater amplitude of the SL-CMJ and thus increased momentum, and could be due to the athlete’s defocusing of the movement towards the dual-task. This finding is noteworthy as athletes with chondropathy and ACL injuries tend to utilize lower dynamic knee flexion angles as a protective strategy against former joint discomfort related to this type of movement, absorbing less energy in the eccentric phase, thus limiting their explosive quality (which is linked to the CMJ’s plyometric aspect) [35]. This suggestion seems to be supported by the fact that greater knee flexion was observed in the healthy control group versus injured peers, which was no doubt linked to the absence of functional deficits and joint damage.

Here, the dual-task reduced trunk flexion in the same proportions for all groups. This effect could be due to the need to process visual information during the CMJ in DT conditions. While additional research is warranted, this visual task may have required that the head and trunk remain as vertical as possible to facilitate the control of the gaze to the pods, i.e., it may have constrained participants to minimize trunk flexion [36]. This strategy of minimizing the trunk flexion would be consistent with the increase in GRFz recorded during the dual-task. Indeed, ACL and CH athletes used higher levels of trunk flexion than CT in order to use hip extension in the propulsion phase and hip flexion when landing, and thus compensate for a lack of knee flexion and deficits in neuromuscular strength and explosiveness [29]. To our knowledge, no study has yet explored the impact of dual-tasking on trunk lateral flexion. However, CH athletes reported higher trunk lateral flexion values compared with the other groups when pushing on the injured leg, presumably in order to compensate for a functional knee deficit [37].

In the present study, activity in nearly all the muscles was increased in the dual-task condition, with the exception of the gluteus medius (no difference) and medial gastrocnemius (decrease) during the pushing phase. Regarding landing, the dual-task activated all the studied muscles more than during the single task, except for the gluteus medius (no difference) and the vastus medialis (decrease). These results are partially in agreement with findings reported by Amoli et al. [38] who reported that adding a dual-task to a CMJ increased the activity of the medial gastrocnemius and biceps femoris and decreased the activity of the rectus femoris and vastus medialis in healthy volleyball players. Here, dual-tasking was related to increased activity in the quadriceps and hamstrings during pushing as well as higher kinetic values. The medial gastrocnemius was less activated owing to injured athletes using more of their thigh muscle to extend their knee during the dual-task. Moreover, quadriceps and hamstring muscles are the most affected (lower activation) muscles by inhibition post-injury [39] due to nerve and structural alterations, which are important risk factors in relapses. The effect of the dual-task was stronger for the ACL group, where muscle activation during pushing and landing was higher than in the other groups, but was only significant in players with a muscle injury. The addition of a dual-task can be pertinent to help obtain a higher activation level of thigh muscle post-injuries to reduce alterations of thigh muscle activation and combat injury recurrence. Our second hypothesis is therefore not entirely validated, as the effects of the dual-task were similar in terms of the kinetic analyses for all groups, while the kinematic impact was more marked for CH, and the increase in muscular activation was greater for ACL versus the other groups.

### 4.3. Effect of the Dual-Task on Post-Injury Performance, Rehabilitation, and Prevention

Finally, despite discrepancies between the present results and those reported in the literature on the negative effect of dual-tasking on motor performance [5,10,32], it seems that the addition of a simple visual information uptake task to a CMJ is beneficial for subsequent jump performance in both healthy and injured athletes. The positive effect of adding a visual task may be due to players’ defocusing of attention onto the secondary visual task rather than on the primary task. Potentially, the secondary task used here was too simple to create a cognitive surcharge in high-level players and, in the end, impacted them more as an external focus (attention concentrated on succeeding the movement). In this case, the results were consistent with other authors’ observations [40] who demonstrated that healthy soccer players improved their jump performance (jump height, rate of force development, concentric impulse) when jump tests were realized with a ball hung high to create an external focus. For injured athletes, this external focusing might have enabled them to ignore any residual pain or fear during the CMJ. The current related literature is contrasting as to whether pain is reduced by dual-tasking or whether dual-tasking is impaired by pain [6,14,29,33].

Athletes with high negative impact of dual-task (medial displacement of the knee, uncontrolled proximal trunk and hip, joint misalignment, and higher loads) during landing or cutting [8,10], particularly during the first 50 ms following ground contact [41], appear to be at risk of incurring non-contact injuries [42]. Indeed, divided attention and decision-making are associated with knee injury [33] during jumping, landing, and cutting tasks [10]. As such, for practical implications, the dual-task must be integrated in neuromuscular training to reduce alterations and modify injury risk factors by automating motor response to the specific sport demands [19]. Dual-task training seems to improve the transfer of motor skills from the conscious control stage to gestural automation [37], leading to greater efficiency [42]. It is thus important that external and internal focusing during motor learning are used by injured athletes [43]. Indeed, staff should integrate dual-task training to improve their athletes’ ability to overcome the limitations of central nervous system processing [37] and respond more safely to the unpredictable aspect of sport and its spatio-temporal, motor pressures and cognitive complexity [37]. They should also increase the complexity of the dual-task for healthy and injured athletes, both in terms of rehabilitation and prevention. If a dual-task becomes too difficult, it could once again impair performance, which could explain the conflicting results in the previous literature. Therefore, appears to be an optimal level of cognitive load [44].

### 4.4. Limitations

This study has some limitations. Firstly, there was no prior protocol registration. The size of the cohort can be considered quite small, although this is common in studies of elite athlete populations. It should be noted that the kinematic assessment equipment has a recording frequency of 30 Hz, which limits the high precision of kinematic analysis. It is difficult to generalize our findings to the entire population because we studied a specific group: elite male footballers.

Finally, the dual-task might have been too simple to create a significant performance alteration. For example, it could be very interesting that players must start, as soon as they receive the ball, on the side indicated by the appearance of an arrow and not a color on a screen at the moment of jumping. This would be more complex and would be closer to the specific footballing gestures used in matches and training sessions.

## 5. Conclusions

This study evaluated the level of motor automation and neuromotor alteration of a typical sport routine movement, in a dual-task, in professional soccer players suffering from various lower-limb injuries compared with healthy peers. The addition of a visual information task during an SL-CMJ movement in high-level soccer players undergoing sport rehabilitation appeared to be a disinhibiting factor, as performance improved, albeit with a deterioration of neuromotor control during the landing phase. A neurocognitive approach is arguably still one of the major missing components in prevention programs [15]. Hence, further work on dual-task inclusion in high-level athletes in the sport rehabilitation and return-to-play phase is necessary [15]. Post-injury athletes tend to maintain compensatory neural strategies, thereby increasing the risk of relapsing, so cognitive-motor work would seem to be a relevant way of limiting this risk [15]. The addition of a dual-task to the various functional return-to-play tests also appears to be an essential area for development in the management of athletes [15] and could also be used as a tool to optimize explosive and jump training during rehabilitation processes.

## Figures and Tables

**Table 1:** Anthropometric features of the different groups of athletes and years spent in a professional league. ACL, MI, CH, CT: groups with anterior cruciate ligament rupture, muscle injury, chondropathy, and control, respectively. Haut du formulaire.

	Height (cm)				Mass (kg)				Age (y)				Years in Professional League			
	ACL	MI	CH	CT	ACL	MI	CH	CT	ACL	MI	CH	CT	ACL	MI	CH	CT
Mean	182	183	183	182	76.5	78	79	75	25.4	25.3	28	24.8	7.1	6.2	7.2	5.7
Std. Deviation	4.1	2	4.4	1.5	7.7	7.5	8.3	7.2	3.8	5.1	2.7	3.6	4	4.5	2.2	3.6
Minimum	175	173	171	168	61.5	63	64	60	19	18	24	20	1	1	4	2
Maximum	189	201	186	192	87	93	89	87	33	34	31	32	15	13	12	14

## Data Availability

The original data presented in the study are openly available in [Database concerns the Effect of the dual task on CMJ.xlsx.] at [DOI: 10.4121/b3cd64a4-3090-4ded-9827-382e64da23a4].

## Supplementary Materials

The following supporting information can be downloaded at: https://www.mdpi.com/article/10.3390/sports13120419/s1, STROBE Checklist—Cross-sectional observational study.

## Abbreviations

The following abbreviations are used in this manuscript:

ACL	anterior cruciate ligament
BF	biceps femoris
CH	chondropathy
CMJ	countermovement-jump
CT	healthy control group
DT	dual task
EMG	electromyography
GM	gluteus medius
GRFz	ground reaction force in the *z*-axis
KF	knee flexion
MG	medial gastrocnemius
MI	muscle injury
MIVC	maximal isometric voluntary contraction
RF	rectus femoris
RFD	rate-of-force development
RSI-Mod	reactive strength index modified
ST	semitendinosus
SLCMJ	single-leg countermovement-jump
TF	trunk flexion
TLF	trunk lateral flexion
TTTO	time to take off
GRFz	vertical ground reaction force
VM	vastus medialis

## Appendix A

### Appendix A.1. Evolution of Kinetic Characteristics

**Table:**

	CH					MI				
	Single Task		Dual-Task		evolution	Single Task		Dual-Task		evolution
	Value	*SD*	Value	*SD*	(%)	Value	*SD*	Value	*SD*	(%)
peak GRFz countermovement (N)	1531	*215*	1589	*223*	3.8	1570	*190*	1579	*230*	0.6
peak GRFz landing (N)	2436	*844*	2377	*780*	−2.4	2727	*731*	2650	*572*	−2.8
RFD eccentric (N/s)	2457	*1121*	2931	*1294*	19.3	2365	*872*	2575	*1184*	8.9
RFD concentric (N/s)	10,871	*2684*	11,694	*2138*	7.6	11,488	*1805*	11,764	*1813*	2.4
RSI-mod (A.U)	0.15	*0.09*	0.19	*0.11*	26.7	0.23	*0.11*	0.24	*0.1*	4.3
Jump height (cm)	7.6	*4.3*	9.1	*4.6*	19.7	12	*4.7*	12.7	*4.3*	5.8
Time to take off (ms)	0.53	*0.13*	0.51	*0.14*	−3.8	0.55	*0.15*	0.56	*0.13*	1.8
Time to peak GRFz landing (ms)	95.6	*16.6*	90.2	*15.6*	−5.6	89.2	*18.6*	86.7	*18.7*	−2.8
	**ACL**					**CT**				
	Single Task		Dual-Task		evolution	Single Task		Dual-Task		evolution
	Value	*SD*	Value	*SD*	(%)	Value	*SD*	Value	*SD*	(%)
peak GRFz countermovement (N)	1488	*196*	1527	*206*	2.6	1617	*256*	1664	*260*	2.9
peak GRFz landing (N)	2362	*585*	2294	*561*	−2.9	2381	*569*	2468	*538*	3.7
RFD eccentric (N/s)	2243	*977*	2517	*1019*	12.2	2977	*1661*	3355	*1686*	12.7
RFD concentric (N/s)	9773	*2111*	10,429	*2196*	6.7	11,749	*2657*	12,019	*2873*	2.3
RSI-mod (A.U)	0.13	*0.06*	0.17	*0.08*	30.8	0.23	*0.08*	0.295	*0.09*	28.3
Jump height (cm)	7.3	*3.8*	8.7	*4*	19.2	11.3	*2.8*	13.7	*2.9*	21.2
Time to take-off (ms)	0.56	*0.13*	0.54	*0.1*	−3.6	0.52	*0.13*	0.48	*0.1*	−7.7
Time to peak GRFz landing (ms)	101.8	*20.9*	98.9	*19.3*	−2.8	90.2	*17*	86	*16.9*	−4.7
