# Supplementary material for: Effect of a Visual Dual-Task on Single-Leg Countermovement-Jump in Male Professional Soccer Players with Lower-Limb Injuries: A Cross-Sectional Observational Study
Source: Sports (Basel). 2025 Dec 1;13(12):419. doi: 10.3390/sports13120419 (PMC12736822; doi:10.3390/sports13120419)
Supplement: Supplementary file 1 [file sports-13-00419-s001.zip › sports-3947861-supplementary.pdf]

STROBE Checklist – Cross-sectional observational study

This checklist was completed in accordance with the STROBE Statement for cross-sectional studies. Each item indicates where it is addressed in the manuscript titled "Effect of a visual dual-task on single-leg countermovement jump in male professional soccer players with lower-limb injuries. *A cross-sectional observational study*"

Item No.	Recommendation	Page/Section (in the manuscript)
1(a)	Indicate the study's design with a commonly used term in the title or abstract.	Title page and Abstract ("A cross-sectional observational study")
1(b)	Provide an informative and balanced summary of what was done and found.	Abstract, p. 1
2	Explain the scientific background and rationale.	Introduction, p. 1–3
3	State specific objectives and hypotheses.	End of Introduction, p. 3
4	Present key elements of study design early.	Methods – Study design, p. 3-8
5	Describe setting, locations, and recruitment period.	Methods – Setting, p. 4
6(a)	Give eligibility criteria and sources/methods of selection.	Methods – Participants, p. 4–5
7	Clearly define outcomes, exposures, and potential confounders.	Methods – Variables, p. 5–7
8	Give sources of data and details of measurements.	Methods – Data recordings & EMG normalization, p. 5–7
9	Describe efforts to address potential sources of bias.	Methods – Bias, p. 7

10	Explain how study size was arrived at.	Methods – Study size, p. 7
11	Explain how quantitative variables were handled in analyses.	Methods – Statistical methods, p. 8
12(a)	Describe all statistical methods, including control for confounding.	Methods – Statistical methods, p. 8
13(a)	Report numbers at each stage (eligible, included, analyzed).	Results – Participants, p. 8-12
14(a)	Give characteristics of study participants.	Table 1, p. 8
15	Report numbers of outcome events or summary measures.	Results sections 3.2–3.4, p. 8-12
16(a)	Give unadjusted and, if applicable, adjusted estimates with precision.	Results, p. 8-12
18	Summarize key results with reference to objectives.	Discussion, p. 12-15
19	Discuss study limitations and potential biases.	Discussion – Limitations, p. 15
20	Provide a cautious interpretation considering objectives and limitations.	Discussion, p. 12-15
21	Discuss generalisability (external validity).	Discussion – Limitations, p. 15
22	Give source of funding and role of funders.	Funding, p. 16
